# The role of metabolism on regulatory T cell development and its impact in tumor and transplantation immunity

**DOI:** 10.3389/fimmu.2022.1016670

**Published:** 2022-12-07

**Authors:** Aleksey S. Bulygin, Julia N. Khantakova, Nadezhda S. Shkaruba, Hiroshi Shiku, Sergey S. Sennikov

**Affiliations:** Laboratory of Molecular Immunology, Federal State Budgetary Scientific Institution Research Institute of Fundamental and Clinical Immunology, Novosibirsk, Russia

**Keywords:** T cells, immunometabolism, glycolysis, fatty acid oxidation, tumor microenvironment, transplant microenvironment

## Abstract

Regulatory CD4^+^ T (Treg) cells play a key role in the induction of immune tolerance and in the prevention of autoimmune diseases. Treg cells are defined by the expression of transcription factor FOXP3, which ensures proliferation and induction of the suppressor activity of this cell population. In a tumor microenvironment, after transplantation or during autoimmune diseases, Treg cells can respond to various signals from their environment and this property ensures their suppressor function. Recent studies showed that a metabolic signaling pathway of Treg cells are essential in the control of Treg cell proliferation processes. This review presents the latest research highlights on how the influence of extracellular factors (e.g. nutrients, vitamins and metabolites) as well as intracellular metabolic signaling pathways regulate tissue specificity of Treg cells and heterogeneity of this cell population. Understanding the metabolic regulation of Treg cells should provide new insights into immune homeostasis and disorders along with important therapeutic implications for autoimmune diseases, cancer and other immune-system–mediated disorders.

## Introduction

CD4^+^CD25^+^FOXP3^+^ regulatory T (Treg) cell subpopulation actively engaged in the induction of immune tolerance to self-antigens and in the control of immune responses (1, 2). By its nature the Treg population is heterogeneous with differences in the expression of transcription factor FOXP3, which is responsible for proliferation processes and immunosuppressive function of these cells. Tregs can be classified into thymic Treg (tTreg or nTreg), *in vitro* generated Treg (iTreg) and Treg induced on the periphery and outside the thymus – peripherally derived Treg (pTreg). In some conditions, such as autoimmune diseases or cancer, Treg cells respond to specific environmental signals and demonstrate plasticity and tissue-specific phenotypes, which help to implement context-dependent suppressive functions (3).

Different states of T-cell activation require the involvement of metabolic signal transduction pathways that are compatible with their function. Recent research indicates that the modulation of metabolism plays an important role in the control of some processes within Treg cells. The transition from lymphocytes of the CD4^+^ lineage to Treg cells is accompanied by active reprogramming of cellular metabolism. Intracellular metabolites and metabolic pathways directly participate in the regulation of FOXP3 expression as well as in the processes of transcription in Treg cells and in their functional plasticity (1, 4). Adaptation of Treg cellular metabolism is required to maintain a functional state in the tumor microenvironment (TME) or after tissue/organ transplantation (5). Such metabolic modifications allow these cells to mobilize all the resources necessary for rapid proliferation and/or full activation of the body’s defense mechanisms as a whole. Thus, cellular metabolism plays an essential part in the regulation of FOXP3 stability expression and Treg cell function.

Thus, the nature of metabolic processes in Treg cells is linked with the induction of a signal for the expression of transcription factor FOXP3. Apparently, this arrangement contributes to specific adaptability of Treg cells in various microenvironmental conditions. Optimization of Treg-based immunotherapies will provide the most accurate understanding of the proliferative, migratory and suppressive behavior of Treg. Recently, it has become obvious that the functional activity of immune cells is directly dependent on the possibilities of metabolism. Treg metabolism can be considered as an important part between the optimization of energy resources and the immune response mechanism, which may indicate the possibility of Treg plasticity between immune response and tolerance (6). This review presents recent research findings about the metabolism of Treg cells and its relation to their regulatory functions.

## Involvement of metabolism in the development of Treg cells

In the thymus, proliferation and development of Treg cells proceed according to the generally accepted mechanism of T-cell development: through the stages of positive and negative selection (7, 8). During the stage of positive selection, the binding of double positive (CD4+CD8+) TCR to a low-affinity peptide–MHC complex located on thymic antigen-presenting cells (APCs), which cooperate with cortical thymic epithelial cells, provides a survival signal for these thymocytes based on the strength of the interaction. Thus, if the interaction is strong the cell will survive to participate in the negative selection process. This interaction of TCR and coreceptor with MHC is necessary to create a population of T cells that can distinguish “self” from “non-self” antigens or cells (9). After positive selection, medullary thymic epithelial cells with dendritic cells produce chemokines CXCL12 and CCL25, which promote the migration of CD4^+^CD8^+^ thymocytes from the cortex to the medulla of the thymus (10). At the stage of negative selection CD4^+^CD8^+^ thymocytes interact through TCR with a high-affinity autopeptide presented by dendritic cells *via* class I and class II MHC complexes. Depending on the avidity of TCR to the antigen, thymocytes either undergo elimination or continue pathway of differentiation. As a result, populations of CD4^+^ T cells or CD8^+^ T cells emerge that have a specific repertoire of TCRs. In this review, we want to focus only on CD4+Treg and how their metabolism develops in general. Only those CD4^+^ T cells become Treg cells whose affinity for class II MHC is stronger than that naive CD4+ effector T cells but below the apoptosis induction threshold (11). The development of thymic regulatory T cells (natural Treg cells: nTreg) proceeds because of FOXP3 expression. The strength and duration of TCR engagement and CD28 costimulation are crucial for FOXP3 expression and for Treg phenotype induction (12). For instance, FOXP3 expression is observed predominantly at the negative selection stage and during the differentiation of CD4^+^ monopositive T cells into nTreg cells. Wherein, there are data on the presence of CD4+CD8+ thymocytes expressing FOXP3 in the thymus cortex in the absence of the TCR signal (13–15). Aside from the TCR signal, the costimulatory pathway mediated by the binding of CD28 of nTreg cells to CD80/86 located on APCs is also important for the modulation of FOXP3 expression. For example, Soskic et al. have demonstrated that positive costimulation of Treg cells results in FOXP3 expression and ensures their homeostasis (16). Thus, it can be deduced that the avidity of the interaction between thymocytes and APCs is a key determinant of Treg cell fate.

Metabolism takes part in the development of nTreg cells *via* a TGF-β–independent mechanism and notably, requires the stimulation of TCR and CD28, which are controlled by the PI3K–AKT–mTOR pathway (17, 18). However, Priyadharshini et al. have demonstrated that, depending on environmental conditions, TGF-β can reprograms Treg metabolism *via* blockade of PI3K–mediated mTOR signaling and glucose metabolism (19). Investigation of key regulators of metabolism-related transcription factors sheds additional light on the function of PI3K–AKT signal transduction in the development of Treg cells. There are four classes of PI3 kinases, but only class IA and class IB PI3Ks have been studied in detail in T cells. The typical object of research is the p110δ class IA catalytic subunit because this protein is predominantly expressed in leukocytes and directly participates in the TCR signal (20–23). p110δ is activated by receptor tyrosine kinases such as cytokine receptors CD25, CD122, CD127, and IFNGR as well as by TCR. p110δ-containing PI3K phosphorylates phosphatidylinositol-4,5-bisphosphate (PIP2) yielding phosphatidylinositol-3,4,5-triphosphate (PIP3) on an inner cell membrane, thereby initiating the activation of downstream signaling factors such as PDK1 and its substrate AKT (21, 24). PDK1 selectively phosphorylates AKT on threonine-308, thus enabling AKT to phosphorylates and inhibits the transcription factor FOXO1, which serves as a positive regulator of Foxp3 expression. This cascade leads to the translocation of FOXO1 from the nucleus to the cytoplasm and to the loss of transcription-regulatory activity by this protein. Overexpression of AKT reduce the number of Treg cells and blocks the development of FOXP3^+^ Treg cells in the thymus (25, 26). Such negative effects are mainly explained by the fact that PDK1 interacts with many other molecules besides AKT, for example, with PKC isoforms (27). Nonetheless, studies focusing on negative regulators of PI3K–AKT signaling, such as the influence of a PH domain leucine-rich repeat-containing protein phosphatase (PHLPP) on PTEN (28), the relation between PI3K and AKT in the development of Treg cells in the thymus. Treg cells express larger amounts of PHLPP1 and PHLPP2 than normal T cells do and thus inhibit the PI3K enzyme, thereby promoting the development of Treg cells in the thymus (29, 30). For example, PHLPP1 knockout mice have a normal number of circulating Treg cells, which means that they may be more dependent on PHLPP2 or less dependent on PI3K–AKT signaling for Treg cell development (31).

**Figure 1:**
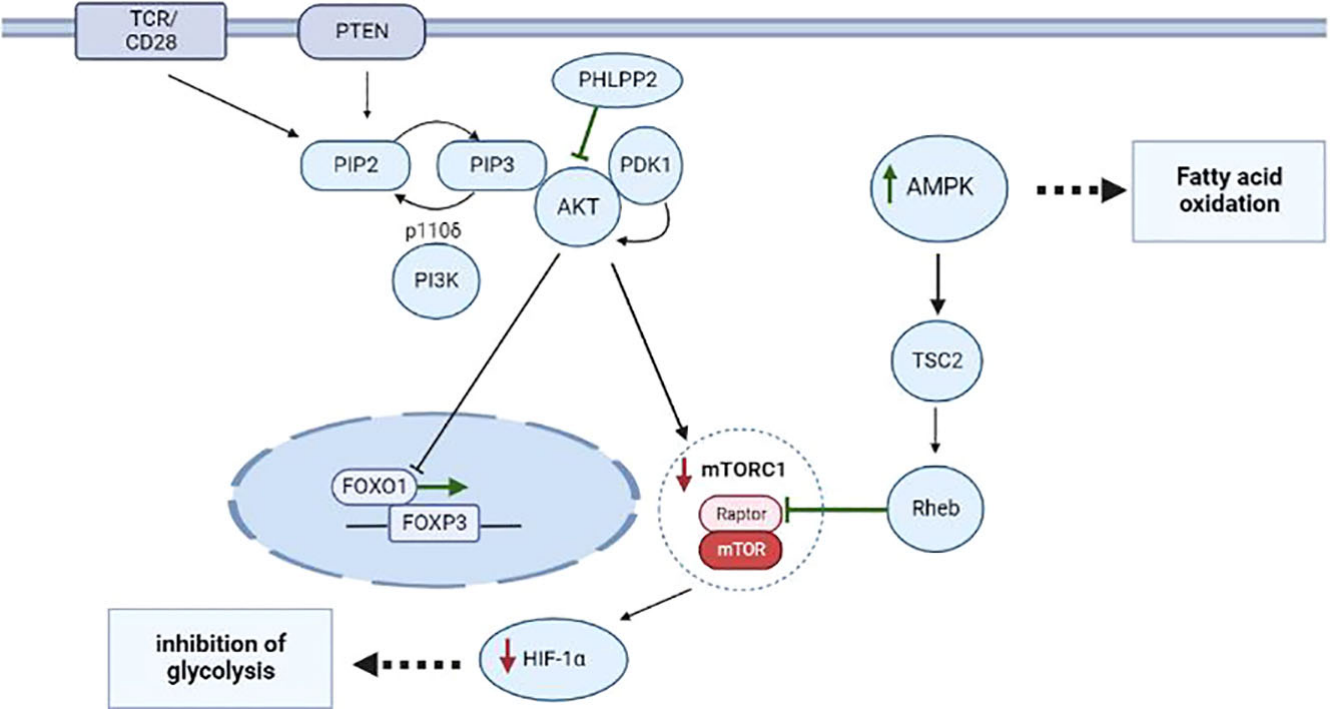
Metabolism of CD4^+^ T cells during proliferation and differentiation into Treg cells thymus and periphery. The most important role in the differentiation of CD4^+^ T cells into Treg cells belongs to the interaction of metabolic and transcription factors during stimulation of TCR and CD28 and activation of the PI3K–AKT–mTORC1 pathway. Additionally, during the negative selection, a phosphatase called PHLPP inhibits the expression of PI3K–AKT pathway components, thus preventing the translocation of FOXO1, thereby upregulating FOXP3 in nTreg cells. *Via* TSC2 and GAP-Rheb, AMPK activity leads to the inhibition of a protein complex known as mTORC1, to suppression of HIF-1α, and to activation of processes responsible for FAO. Events that induce differentiation into Treg cells are highlighted in green; processes that are inhibited in Treg cells are marked in red.

The PI3K–AKT signaling cascade is connected with one of the key factors of metabolism: mammalian target of rapamycin complex 1 (mTORC1). mTORC1 is a key node for the integration of metabolic signals and promotes anabolic processes during cell proliferation and differentiation; subsequently, mTORC1 serves as a target of AKT (32, 33). At the positive selection stage, CD4^+^CD8^+^ T cells overexpress glucose transporter 1 (GLUT1). Presumably, glycolysis is required to a greater extent for the support of cell growth and for meeting proliferation-related needs of the cells. Multiple glycolytic enzymes are targets of AKT and the PI3K–AKT–mTOR pathway, as reported, promotes the transfer of GLUT1 to the cell surface (34, 35). Conversely, during the negative selection stage when CD4^+^ T cells differentiate toward Treg cells, T cells become more dependent on high intensity of lipid oxidation for their survival (36–38). Compared to CD4^+^ and CD8^+^ T cells, in the Treg population, GLUT1 is underexpressed, and blockade of PI3K or mTORC1 reduces glycolysis in T cells and promotes the differentiation into Treg cells in the thymus and in the periphery (**Figure 1**) (39). For example, inhibition of mTORC1 causes a change in Treg cell metabolism towards increased lipogenesis and of the mevalonate-dependent pathway of cholesterol biosynthesis intended to maintain the proliferation and functioning of Treg cells (40, 41). In this context, excessive mTORC1 activation and unlimited glycolysis in Treg cells are characterized by low FOXP3 expression accompanied by a decline of the suppressive activity of Treg cells (42, 43).

According to the above-mentioned data, 5′AMP-activated protein kinase (AMPK) is responsible for reducing the expression of GLUT1 and mTORC1 (44). AMPK is a serine/threonine protein kinase consisting of a catalytic subunit (AMPKα) and two regulatory subunits (AMKPβ and AMPKγ), and its function is to respond to substantial energy consumption and to regulate the energy balance in the cell (45, 46). AMPK is activated when intracellular adenosine triphosphate (ATP) production diminishes and intracellular adenosine monophosphate (AMP) concentration goes up, as seen during nutrient deprivation or hypoxia (45). A recent research article revealed that AMPK is activated in T cells when they are stimulated (e.g. cytokines, TCR, or other factors) and plays an important part in T-cell metabolism (47). For example, Zhu et al. have shown that upon stimulation of TCR, AMPK activation triggers FOXP3 phosphorylation by inhibiting E3 ubiquitin ligases and proteasomes *in vitro* and *in vivo* (48). AMPK acts through phosphorylation of tuberin (TSC2), thereby launching a cascade of mTORC1-inhibitory reactions (49). TSC2 is a GTPase-activating protein (GAP) that participates in the regulation of energy flow during growth and stress and interacts with mTORC1 signaling through the GTP– bound Rheb (50). Phosphorylation of TSC2 on threonine-1227 and serine-1345 by AMPK activates TSC2, leading to the formation of a large amount of the GTP– bound Rheb, thereby shifting the balance toward the inhibition of activity of a substrate called Raptor in mTORC1 (49, 51). Inhibition Hypoxia-inducible factor 1 alpha (HIF-1α) a lead to the induction of the Treg phenotype in T cells at the negative selection stage (**Figure 1**). Recent research suggests that Treg cells also utilize glucose. In particular, it was demonstrated that Treg cells have a large amount of a glycolytic enzyme called enolase, which is involved in *FOXP3* splicing during the formation of pTreg cells at the periphery *in vivo* (52).

Treg cells show both high magnitude of fatty acid β-oxidation (FAO) and elevated expression of genes taking part in the regulation of FAO (53, 54). An important component of the regulation of fatty acid metabolism is carnitine palmitoyl transferase 1A (CPT1A). Treg cells harboring large amounts of activated AMPK along with characteristic FAO, probably due to a specific feature of AMPK, are able to modulate CPT1A activity and increase mitochondrial fatty acid import for β-oxidation. CPT1A is a protein found in the outer mitochondrial membrane and catalyzes the esterification of long-chain acyls by carnitine producing acylcarnitine; CPT1A is thought to be a regulator of the rate of long-chain fatty acid oxidation (55). On the other hand, studies showed that FOXP3 expression in Treg cells alters metabolic pathways for the oxidation of long-chain fatty acids, thus making Treg cells stable under some conditions (56, 57). FOXP3^+^ Treg cells are also known to take up long-chain fatty acids *via* CD36 as a receptor. Short- and medium-chain fatty acids passively diffuse through the cytoplasm and mitochondrial membranes to participate in FAO (58, 59). This means that some of the upregulated oxidative phosphorylation (OXPHOS) mediated by FOXP3 is linked with oxidation of exogenous fatty acids. These cells also simultaneously increase own expression of genes coding for complexes of the mitochondrial electron transport chain. Taken together, these data mean that the regulation of metabolic pathways is closely connected to T-cell development along the Treg lineage.

Depending on the immunosuppressive function of Treg cells, metabolism can be reprogrammed between glycolysis (60) and OXPHOS (61). HIF-1α can participate in regulation cytokine expression Treg. Groneberg et al. have demonstrated that HIF-1α induces IL-2 and Foxp3 expression in Treg, resulting in increased expression of IL-10 in Tregs (62). In the periphery, PD-L2 costimulation maintains Treg immunosuppressive activity by inducing CPT1A expression. Induction of CPT1A leads to the enhancement of OXPHOS process due to the stabilization of the TCA cycle, this promotes the active production of acetyl-CoA, which in its turn, leads to an increase in the expression of IL-10 pTreg (63). Resent research suggest that IL-35 plays an important role in regulating Treg metabolism. For example, inhibition of IL-35 expression leads to a change in the metabolic activity of Treg cells with an increased glycolysis and production of effector cytokines (64). In addition, the importance using of extracellular nutrients can influence Treg plasticity. For example, I. Matias et al, has shown that the addition of α-ketoglutarate led to a reprogramming of metabolism towards OXPHOS with storage lipids, which led to Treg differentiation with an inflammatory phenotype, manifested by increasing production of cytokines IFN-γ and granzyme B (65).

It can be concluded that metabolism has a significant role in the proliferation and development of Treg cells in the thymus and periphery. Moreover, this phenomenon manifests itself in the characteristic functioning of glycolysis, which promotes the flux of biomolecules in synthesis for the differentiation of CD4^+^ lymphocytes into Treg cells. Subsequently, the transition to the consumption of alternative energy sources according to the FAO mechanism is observed in the formed Treg. In this context, the strength of the signal from TCR and CD28 and the regulation of the PI3K–AKT–mTORC1 cascade are critical for FOXP3 expression and for the formation of Treg cells in general.

## Metabolism of Treg cells during tumor growth as well as after transplantation

So far, investigation into such characteristics of Treg cells as adaptation to a microenvironment and to systemic changes remains a relevant and important scientific task. Treg cells can be considered a specific subclass of the CD4^+^ T-cell pool. Because they are physiologically present in almost all tissues and organs, while expressing a wide range of immunological and metabolic markers. Therefore, Treg cells are able to quickly respond to any immunological and metabolic signals under the conditions of tumor infiltration or after a tissue or organ transplant. Owing to Treg cells’ high sensitivity to external signals and their fundamental role in the switching between tolerance and immunity, metabolism can be regarded as one of the key mechanisms behind the regulation of Treg cells. In the following section, we describe the dynamics of metabolism in the regulation of Treg cells under conditions of tumors and transplantation.

**Figure 2:**
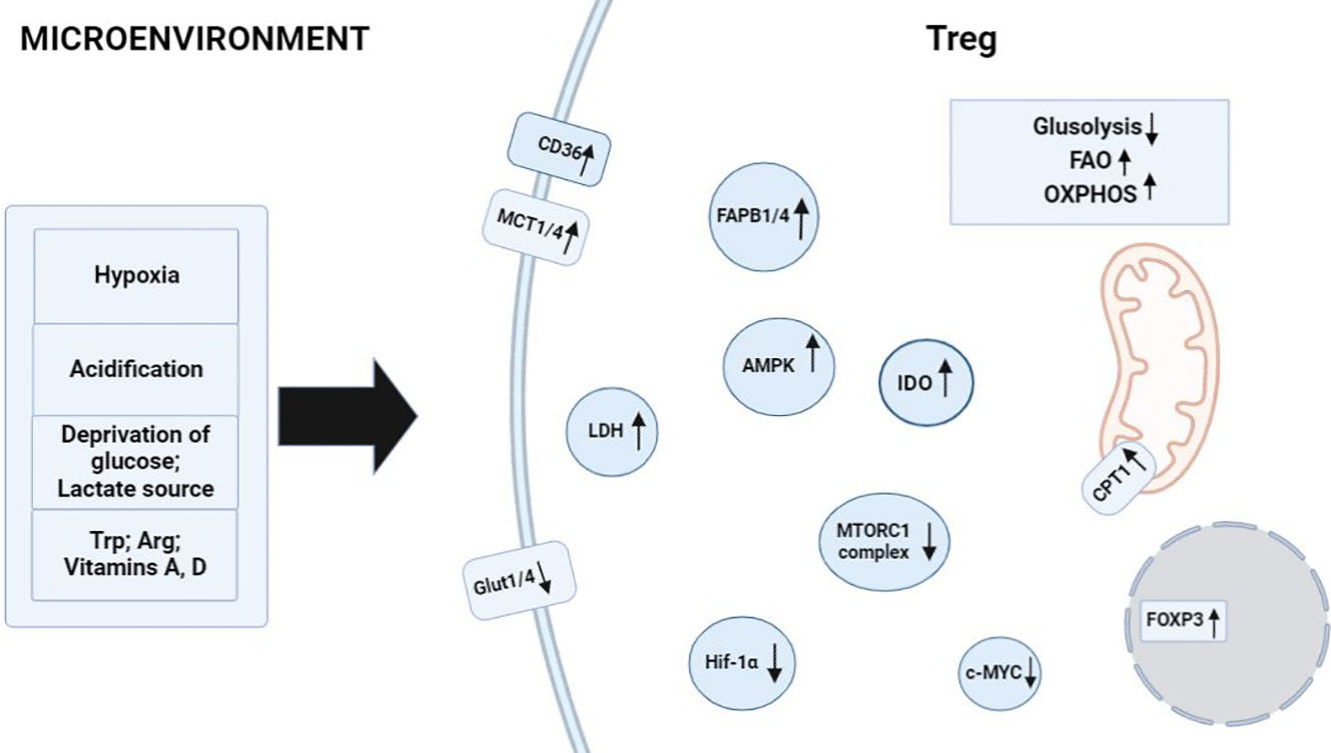
Factors in the microenvironment that contribute to metabolism in Treg cells. Activating environmental signals are required for functional maturation of CD4^+^ T cells into Treg cells and induce processes related to the regulation of FAO and OXPHOS. Hypoxia and acidification of the environment raise the number of Treg cells owing to their specificity for FAO processes. Glucose depletion and a high concentration of lactate in the medium cause the activation of enzymes AMPK, CD36, MCT1 or MCT4, FABP1 or FABP4, and CPT1 in Treg cells, thereby promoting their full-fledged physiological activities in the microenvironment. Tryptophan catabolism and arginine catabolism enhance IDO enzymatic activity in FOXP3^+^ Treg cells. TGF-β and IL-10 induce the formation of Treg cells and drive the reprogramming of metabolic pathways toward FAO. ↑: processes and biomolecules that are upregulated in Treg cells under the influence of microenvironmental factors; ↓: processes and biomolecules that are downregulated in Treg cells when the latter are exposed to microenvironmental factors.

The study of Tregs metabolism in tumors and transplantation is important due to negative effect of Tregs in immunotherapy. The interaction between tumor cells and Treg promotes their favorable cohabitation and population growth. During transplantation the conditions for the growth of Tregs are unfavorable, which is expressed by their small number in the environment and the occurrence of an immune response to the transplant (**Figure 2**). In this regard, the study of Treg metabolism under the influence of tumors and transplantation will contribute to the development of new approaches in immunotherapy and transplantation.

### Treg cells during tumor growth

**Figure 3:**
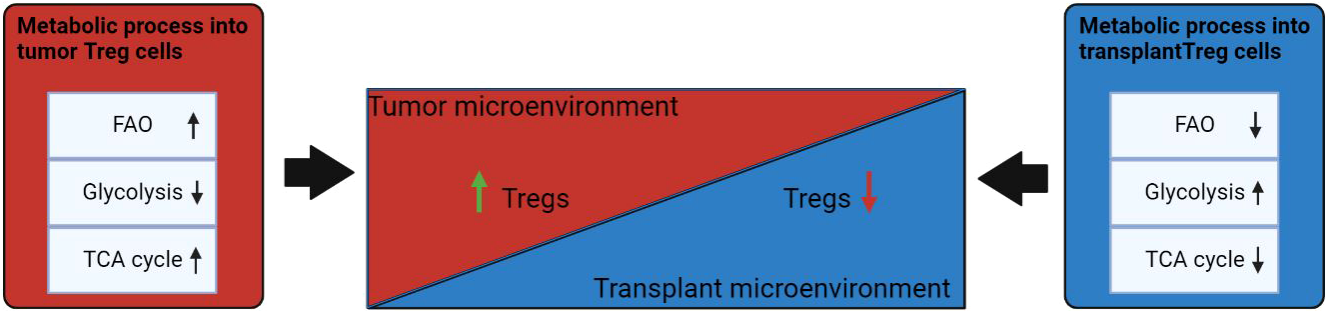
Influence of the microenvironment on the metabolism of Treg cells. Under conditions of TME, FAO and TCA cycles are increased in Tregs. In the transplantation microenvironment, Treg has a reduced fatty acid metabolism and a transition to glycolysis uptake of alternative energy sources. Events that induce differentiation into Treg cells are highlighted in green; processes that are inhibited in Treg cells are marked in red. ↑: metabolic processes that are upregulated in Treg cells under the influence of microenvironmental factors; ↓: metabolic that are downregulated in Treg cells under influence of microenvironmental factors.

Dysregulation of cellular energy mechanisms—including enhanced aerobic glycolysis, *de novo* fatty acid synthesis, and glutaminolysis—satisfies cancer cells’ energy requirements and proliferation-related needs. For example, increased glycolysis and lactate production by cancer cells help to protect the tumor from an immune response. However, it create an immunosuppressive TME by depriving infiltrating effector T cells of nutrients and by “providing” immunosuppressive metabolites (66–68). In contrast to helper and cytotoxic T cells, which primarily use aerobic glycolysis and anabolic processes to satisfy own biological energy needs, Treg cells are believed to rely on FAO *via* OXPHOS to maintain their differentiated state and activities (**Figure 2**) (69–71). Consequently, in the TME, glucose depletion, which is detrimental to a variety of CD4^+^ and CD8^+^ T cells, may have a negligible effect on Treg cells (**Figure 3**). Metabolic adaptation in the TME promotes the survival of Treg cells in an aggressive environment. Recent study suggests that Treg cells underexpress membrane glucose transporter GLUT1 and show increased lipid oxidation activity as compared to other effector T-cell subpopulations. Effector T cells and Treg cells exhibit different metabolic patterns: the former require glycolysis, whereas the latter require lipid oxidation for survival and functioning (69, 72, 73). This metabolic preference is regulated by the expression of the Treg-specific transcription factor FOXP3, which additionally inhibits glycolytic enzymes and downregulates the master regulator c-Myc (74). Furthermore, tumor cells to convert pyruvate to lactate *via* lactate dehydrogenase (LDH) in a process called aerobic glycolysis (Warburg effect), which is a potential nutrient for tumor Treg. Upon uptake of lactate and lactic acid, LDH catalyzes their reversible conversion to form pyruvate and NADH. Tregs have also been found to uptake LDH from the extracellular space *via* monocarboxylate transporter 4 (MCT4) to support own differentiation and survival *in vitro* (75–77). It should be noted that high lactate uptake disturbs aerobic glycolysis and forces Treg cells to perform OXPHOS. Apparently, the high aerobic glycolytic activity of tumor cells can create a microenvironment in which glucose deprivation and lactic acid enrichment promote the survival of Treg cells. It helps maintain the immunosuppressive function of the Treg cells that are in contact with the tumor. For example, Angelin et al. have shown that the specialized metabolic preferences of Treg cells support their survival in an environment enriched with lactic acid but devoid of glucose (74).

Latest studies on the suppressor function of Treg cells in the TME have revealed that the functionality of Treg cells is affected by several metabolic signals. In this regard, a hypoxic environment enhances the suppressor function of Treg cells. As demonstrated in a mouse glioma model, stabilization of HIF-1α in Tregs balances Treg migration (positively regulated by aerobic glycolysis) with Treg suppressive ability (positively regulated by mitochondrial OXPHOS of FAs) to ensure tumor growth. To do so, HIF-1α sustains both aerobic glycolysis and FAO (OXPHOS of FAs) by activation of lipid transporters [CD36 and fatty acid-binding proteins 1 and 4 (FABP1 and FABP4) (**Figure 3**) (38, 78). Elevated lipid uptake is key to the strength of Treg suppressive function, which manifests itself as a reduction in CD8^+^ T-cell proliferation during low HIF-1α expression (**Figure 3**) (78). In this context, treatment with etomoxir, a mitochondrial CPT1/FAO inhibitor, enhances antitumor immunity by augmenting the abundance of CD4+FOXP3+ T cells while curtailing their immunosuppressive capabilities (78). Recent investigation revealed that rapid glucose metabolism alters the functionality of Tregs; in a study on human tumors, glycolytic genes, including hexokinase 2 (*HK2*), glyceraldehyde 3-phosphate dehydrogenase (*GAPDH*), and α-enolase (*ENO1*), were found to be overexpressed in Treg cells (72). Consequently, it is possible that Treg cells under the conditions of contact with tumor cells are capable of metabolic adaptation, which opens up opportunities for differentiation of Treg cells and for inhibition of their suppressor activity toward effector T cells.

Many strategies are currently being used to manipulate Treg cells, including depletion of Treg cells, inhibition of Treg cell function, or blockade of Treg cell recruitment to lymph nodes or tumors (79). For example, inhibition of FABP5 function on the surface of Treg cells has been demonstrated, which attenuates OXPHOS and thus eventually impairs lipid metabolism and causes a loss of proper structure of mitochondrial cristae *in vitro* and *in vivo*. Destruction of FABP5 triggers the release of mitochondrial DNA into the cytosol, activating the cGAS-STING-dependent type I IFN signaling pathway, which upregulates Treg suppressive activity through enhancing IL-10 production (38). Another research demonstrated that activation of Toll-like receptor 8 (TLR8) signaling in Treg cells suppresses metabolic processes of glycolysis, thus ultimately weakening the suppressor function of Treg cells *in vitro* and *in vivo* (80). Further research aimed at the regulation of metabolic signaling pathways in Treg cells may point to a new strategy for cancer prevention or treatment.

Thereby, the microenvironmental conditions created by tumor cells contribute to the survival of Treg cells and to enhancement of their suppressor activity, which is manifested in the inhibition of antitumor immunity. This feature is based on the capacity of Treg cells for OXPHOS *via* maintenance of the activity of CD36 or FABP1 and FABP4, whereas effector T cells have to compete for glucose with tumor cells. In the latest studies, pharmacological and genetic interventions into the key transcriptional mechanisms of fatty acid metabolism in Treg cells help to increase the effectiveness of therapy in combination with existing antitumor strategies.

### Treg cells after transplantation

Metabolism of Tregs in case of transplantation slightly differs in comparison to the conditions in TME that contribute to the induction of immune tolerance. The delicate balance between aerobic glycolysis and OXPHOS in Treg cells is controlled by mTOR and AMPK signaling pathways, respectively, thus linking immunological and microenvironmental factors to Treg cell metabolism and function after transplantation (**Figure 3**). FOXP3, in combination with the activation of TCR, IL-2 and mTOR, play a key role in the enhancement of Treg cell proliferation and function in allotransplantation settings. Several studies have demonstrated how manipulation of these metabolic factors can in turn modulate the function of Tregs. This is important because peripheral Treg cells must survive and maintain tolerance upon transplantation (81). During inflammation associated with allograft rejection the microenvironment is infiltrated by effector T cells, which begin to compete with Treg cells on metabolites for survival and induction of an immune response, which ultimately leads to a decrease in Treg (82).

In transplant settings Treg cell metabolism is also known to be associated with FAO and OXPHOS as described above in TME. Additionally, activated Treg cells can utilize extracellular glutamine as an alternative source of energy. Indeed, glutamine can be imported, incorporated directly into the tricarboxylic acid cycle and catabolized *via* OXPHOS in a process called glutaminolysis. Glutaminolysis provides all types of T cells with carbon and nitrogen necessary for the biosynthesis of hexosamine and nucleotides and is an important source of materials for T-cell proliferation. At high glutamine concentrations FOXP3 expression is induced, and differentiation toward the T helper 1 (Th1) phenotype is weakened (83, 84). For instance, oral glutamine supplementation increases the number of circulating Treg cells and alleviates graft-versus-host disease in mice compared with controls (85). Other metabolites that may influence Treg cell function are vitamins A and D, tryptophan, and arginine (86–88). Retinoic acid (a bioactive vitamin A metabolite) promotes differentiation of naive T cells into FOXP3^+^ Treg cells (**Figure 2**) (86). Retinoic acid stabilizes FOXP3 expression and prevents the differentiation of Treg cells under the action of IL-1β/IL-6 into Th1/Th17 cells (89). An active metabolite of vitamin D called calcitriol increases the proportion of IL-10-producing Treg cells upon transplantation (87, 90). Addition L-arginine increases IL-10 production of Tregs through IL-10 promoter DNA hypomethylation (91). Expression of indoleamine 2,3-dioxygenase (IDO) promotes the catabolism of L-tryptophan to kynurenine, a compound that is important for the induction of the Treg phenotype (**Figure 2**) (88). In several transplantation models, increased IDO activity in transplanted cells has been found to exert antirejection effects *in vitro* and *in vivo* (88, 92). For example, it has been demonstrated that most of the co-stimulation-blocking effect of CTLA4 is mediated by elevated IDO activity in dendritic cells. This discovery, together with the ability of IDO-competent dendritic cells to induce the Treg phenotype in T cells, implies the existence of a peripheral tolerogenic pathway with important implications for transplantation medicine (93). Besides, after a skin transplant dendritic cells consume various essential amino acids to synthesize IDO, which ultimately leads to a decrease in mTOR activity and to induction of FOXP3 in T cells (94). To sum up, in transplantation settings, Treg cell metabolism responds to external signals (not only to its own “needs” and presented conditions in the microenvironment): for example, to metabolites of vitamins A and D or of cells that can affect Treg cells.

The therapeutic use of Treg cells for control over an unwanted immune reaction is an active area of ​​research. In the last few years studies were published about a strategy to regulate the metabolic reprogramming of Treg cells in transplantation therapy. For instance, treatment with coenzyme Q10 has been shown to restore the activity of OXPHOS in pSTAT3-inhibited iTreg cells *in vitro* and *in vivo*. This approach allows to enhance their suppressive activity against alloreactive T cells after a skin transplant (95). In addition, Treg survival during inflammatory reactions, such as during allotransplantation, may be mediated by Notch expression. Saini et al. have demonstrated that activation of signal pathway Notch1-Grp75 maintains calcium ion homeostasis in mitochondria, which finally ensures the operation of oxidative phosphorylation in Treg cells (96). In general, there is a trend of growing importance of the research into the regulation of Treg cell metabolism in transplantation therapy in order to expand the list of options for overcoming the rejection reaction and autoimmune diseases.

Overall, latest research findings show the importance of understanding regulation the metabolism flux of Treg cells in transplantation settings. The induction of the Treg phenotype contributes to effective suppression of immune responses in models of graft-versus-host disease and of organ transplantation. Research in recent years will provide a better understanding of the efficacy of Treg cell therapy in humans.

## Concluding remarks and future perspectives

The field of immunometabolism research and the regulation of Treg cells, in particular, is developing rapidly. Additional opportunities are arising for the manipulation of potentially useful features of Treg cell metabolism under various conditions. Nevertheless, there are still many unresolved issues. For example, the maintenance of a balance between metabolism and immunological function in Treg cells is a complex process of interrelations with a constant dependence on environmental conditions. Furthermore, investigators have yet not fully formulated the concept of switching of Treg cells from “classic” aerobic glycolysis to an alternative energy source in the form of OXPHOS *via* FAO. Recent advances in immunotherapy have focused on the checkpoint molecules of Tregs or the adoptive transfer of effector T cells. For instance, the metabolic potential of CAR-T cells and T effector cells could be enhanced to overcome the harmful effects of the TME and enhance their anti-tumor aptitude (97). In transplantation, induction of liver kinase B1 (LKB1) signaling may promote Treg survival and function (98, 99). Active research is underway regarding the regulation of metabolism during intercellular interactions in a microenvironment and regarding effects of metabolism stimulation on the suppressor function of Treg cells under various conditions, e.g., during tumor growth or after transplantation.

Technological advances, such as high-throughput RNA sequencing, are likely to provide complete information about the regulation of metabolic reprogramming of Treg cells in various situations. Present-day methods enable scientists to study genetic and pharmacological modulation of metabolic signaling pathways in Treg cells. This approach paves the way to research on the mechanisms of interaction of intracellular signals and lays the foundation for the development of new applications of Treg cells in therapy. Particularly, this knowledge will be needed in clinical practice to implement new modalities for the treatment of various diseases.

## Author contributions

AB wrote the manuscript. JK prepared the figure. NS prepared the figure. HS planned and supervised the part of cancer study. SS planned and supervised the part of transplantation study. All authors contributed to the article and approved the submitted version.

## Glossary

**Table:**

AKT	protein kinase B
AMP	adenosine monophosphate
AMPK	5′AMP-activated protein kinase
APC	antigen-presenting cell
ATP	adenosine triphosphate
CPT1A	carnitine palmitoyltransferase I
ENO1	α-enolase
FABP	fatty acid-binding protein (transporter)
FAO	fatty acid β-oxidation
FOXO1	forkhead box protein O1
FOXP3	forkhead box P3
GAP	GTPase-activating protein
GAPDH	glyceraldehyde-3-phosphate dehydrogenase
GLUT1	glucose transporter, type 1
HIF-1α	hypoxia-inducible factor 1 alpha
HK2	hexokinase 2
IDO	indolamine 2,3-dioxygenase
LDH	lactate dehydrogenase
MCT4	monocarboxylate transporter 4
MHC	major histocompatibility complex
mTORC1	mammalian target of rapamycin complex 1
NADH	reduced nicotinamide adenine dinucleotide
nTreg cells	natural regulatory CD4^+^ T cells
OXPHOS	oxidative phosphorylation
PDK1	phosphoinositide-dependent protein kinase 1
PHLPP	PH domain leucine-rich repeat-containing protein phosphatase
PI3K	phosphoinositide 3-kinase
PIP2	phosphatidylinositol-4,5-bisphosphate
PIP3	phosphatidylinositol-3,4,5-triphosphate
PKC	protein kinase C
Rheb	GTPase-binding protein
TCR	T-cell receptor
Th	helper CD4^+^ T-cell phenotype
TME	tumor microenvironment
Treg cells	regulatory CD4^+^ T cells
TSC2	tuberin
